# Feasibility of improving child behavioral health using task-shifting to implement the 4Rs and 2Ss program for strengthening families in child welfare

**DOI:** 10.1186/s40814-016-0062-2

**Published:** 2016-05-02

**Authors:** Geetha Gopalan

**Affiliations:** University of Maryland School of Social Work, 525 West Redwood Street, Baltimore, MD 21201 USA

**Keywords:** Task-shifting, PRISM, 4Rs and 2Ss for strengthening families program, Multiple family groups, Child behavior difficulties, Child welfare, Cross-setting implementation

## Abstract

**Background:**

Children whose families are involved with child welfare services manifest disproportionately high levels of behavioral difficulties, which could be addressed in community-based organizations providing services to prevent out-of-home placement. Unfortunately, few evidence-based practices have been successfully implemented in child welfare settings, especially those originally delivered by mental health providers. Given that such settings typically employ caseworkers who lack prior mental health training, this is a significant barrier to implementation. Consequently, the overall aim of the current study is to test the feasibility of shifting a mental health intervention from specialized services to community-based organizations. It uses task-shifting and the Practical, Robust, Implementation, and Sustainability model (PRISM) to implement an evidence-based intervention to reduce child behavior difficulties, originally provided by mental health practitioners, so that it can be delivered by caseworkers providing placement prevention services to child welfare-involved families. Task-shifting involves (1) modifying the intervention for provision by non-mental health providers, (2) training non-mental health providers in the modified intervention, and (3) establishing regular supervision and monitoring by mental health specialists.

**Methods/design:**

This study uses the 4Rs and 2Ss Program for Strengthening Families, a multiple family group service delivery model to reduce child behavior difficulties, as the example intervention. This intervention has had prior success with child welfare-involved families. The proposed study objectives are (1) to tailor the content, training, and supervision of the intervention for delivery by caseworkers serving child welfare-involved families and (2) to assess the feasibility and acceptability of the modified intervention. Mixed quantitative and qualitative methods will assess feasibility and acceptability from key stakeholders (caseworkers, supervisors, administrators, caregivers). In phase I, a collaborative advisory board will be convened (1) to modify the intervention to be delivered by caseworkers in placement prevention service settings and (2) to develop training and supervision protocols for caseworkers. In phase 2, the modified intervention will be pilot-tested for delivery by *n* = 4 caseworkers to *n* = 20 families receiving placement preventive services (where children manifest behavior problems). Mixed quantitative/qualitative methods will be used to assess feasibility and acceptability.

**Discussion:**

This protocol will be of particular interest to agency administrators, program managers, and researchers interested in developing and testing cross-setting implementation guidelines for similar evidence-based practices.

## Background

Children who remain at home with their permanent caregivers following a child welfare investigation manifest disproportionately higher rates of behavior problems (e.g., hyperactive, oppositional, disruptive, and/or aggressive behavior [1]) compared to national rates [2, 3]. The current study focuses on this subgroup as it represents the majority of youth involved in child welfare services within the USA [4] but who are less likely to utilize child mental health services compared to youth placed into foster care [5]. Untreated, such behavioral problems are a costly (up to tenfold increase) [6] public health concern as they also create additional service needs due to increased risk for future maltreatment [7, 8], out-of-home placement [9], high school dropout [10], and delinquency [11]. Effective evidence-based practices (EBPs), such as behavioral parent training interventions [12, 13] known to successfully reduce child behavior difficulties, are needed particularly for those families who are identified as high risk, but where there remains the possibility of averting a traumatizing out-of-home placement, as well as reducing the risk of other future maladaptive outcomes.

Many such behavioral parent training programs are offered in child mental health settings. However, reduction of maltreatment risk and improving family stability through parent training fall well within the purview of community-based organizations (CBOs) which provide a comprehensive array of placement prevention services (also known as “in home family preservation,” “preventive services”) for families mandated or referred by child welfare authorities following maltreatment investigations [14, 15] as well as a small proportion of families with similar difficulties voluntarily seeking services [16]. Rather than refer families to separately housed (and often overburdened [17]) child mental health providers, CBOs are logical platforms for effective interventions to reach child welfare-involved children with behavior problems. At the same time, many such EBPs were originally designed to be delivered by advanced mental health providers (e.g., Parent-child interaction therapy [13]), a substantial implementation barrier, given that the typical child welfare workforce includes a sizable proportion of caseworkers lacking advanced specialized mental health training [18, 19].

Despite recent efforts to implement EBPs developed for small clinics in child welfare settings [14, 20], there is limited guidance available about successful strategies for implementing EBPs from one service setting to another [21, 22]. In addition to lacking ancillary psychiatric support or providers with extensive mental health backgrounds, caseworkers have sizeable workloads and multiple responsibilities, with few available avenues to support knowledge sharing [23, 24]. CBOs also differ from child mental health settings with regard to perceived roles and responsibilities among staff, consumer characteristics, fiscal and regulatory requirements, and the capacity of organizations to integrate innovation into practice as well as state-specific licensure and certification laws which may prohibit formal mental health treatment by non-licensed individuals. As a result, modifying intervention processes to align with caseworkers’ scope of practice may be necessary as well as subsequent evaluation of the adapted model’s feasibility, acceptability, and effectiveness [22, 25]. In addition, partnering with community stakeholders (e.g., consumers, providers) and EBP experts around adapting intervention processes and materials, increasing agency capacity, and developing collaborative partnerships can leverage differing stakeholder competencies in the service of promoting successful implementation and outcomes [21, 22, 26].

The current study attempts to increase guidance regarding cross-setting adaptations and implementation processes. Specifically, task-shifting strategies [27] and the Practical, Robust, Implementation, and Sustainability Model (PRISM [28]) are utilized to modify the 4Rs and 2Ss for strengthening families program (4R2S), an evidence-based multiple family group service delivery model to reduce child behavioral difficulties, for implementation in CBOs serving families involved in placement prevention services. The modified intervention will subsequently be pilot-tested for feasibility and acceptability.

### Task-shifting

**Fig. 1:**
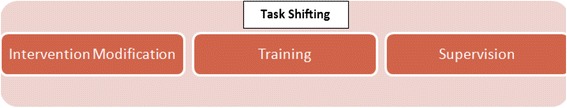
Task-shifting strategies. Successful task-shifting efforts to increase access to mental health treatment which in the developing world involve (1) modifying the original intervention so that it can be delivered to non-mental health specialists and in the contexts within which they work; (2) training non-mental health specialists in the modified intervention, and (3) providing regular supervision with a mental health specialist [29–32]

Endorsed by the World Health Organization (WHO) for use by developing countries with shortages in mental health professionals, task-shifting involves redistributing tasks from professionally trained workers to those with less training and fewer qualifications [27]. As illustrated in Fig. 1, successful task-shifting efforts [29–32] in the developing world have involved (1) modifying the intervention to be delivered by non-mental health workers in their existing settings (e.g., simplifying language, length, service delivery modality), (2) training non-mental health workers, and (3) providing consistent supervision for task-shifted non-mental health workers by competent mental health providers. As a result, task-shifting strategies have successfully reduced adult depression using Cognitive Behavior Therapy and Interpersonal Psychotherapy in Pakistan, Uganda, India, and South Africa by a host of lay-level providers [29, 33–35]. Most importantly, the beneficial effects were achieved by continuously supervised [35] workers with no mental health background and relatively short training (as little as 2 days [31, 32, 34]). Within the USA, task-shifting from highly trained professionals to providers with less training and experience has been used in efforts to improve dental care in rural settings (NIH Grant R36HS019117-01A1), promote healthy body image among college undergraduates [36], provide behavioral parent training for low-income families in Head Start [37], and increase access to services for aging individuals with dementia [38] as well as numerous efforts shifting tasks from physicians to lesser-skilled health professionals (e.g., nurses) to increase access to medical services [39–42]. To the author’s knowledge, task-shifting has yet to be used to implement EBPs from child mental health to the child welfare service sector, where mental health specialists are not typically employed. In the current study, task-shifting will provide an overarching conceptual framework to translate mental health clinicians’ skills and strategies so that they can be used by caseworkers while maintaining compliance with licensure-related restrictions on scope of practice.

### PRISM

Specific modifications to the intervention, development of training and supervision protocols, and measurement will be further guided by PRISM, a practical, robust, and prescriptive model built upon multi-disciplinary implementation models. Table 1 documents the PRISM domains and elements of interest to maximize implementation success and identifies potential areas of modification for the current study. PRISM emphasizes multiple domains that should be considered in order to promote implementation success. Specifically, PRISM domains will specify aspects within the task-shifting approach (i.e., intervention modification, training, and supervision) that will be addressed to support implementation success across CBOs. Moreover, PRISM domains guide measurement of feasibility and acceptability in the proposed study. Although the PRISM framework has been frequently cited within the implementation literature as informing studies, the author is unaware of any studies which utilize PRISM as a conceptual framework informing implementation in the child welfare service sector. Moreover, PRISM is utilized in the current study as a framework for intervention adaptation into a new setting such that the resulting modified 4R2S intervention is maximized for subsequent implementation success. Such use of an implementation framework may be increasingly necessary to guide basic intervention development in order to increase the likelihood that newly developed interventions are integrated into everyday practice. This study focuses on PRISM domains emphasizing intervention perspectives, recipient characteristics, and the external environment. Once feasibility and acceptability are established, the next stage in this line of research will be to conduct a larger-scale study incorporating multiple CBOs, addressing the PRISM domain focused on implementation and sustainability infrastructure, and examining implementation and intervention effectiveness outcomes.

**Table 1:** PRISM domains, elements, and anticipated modification/development focus

PRISM domains	PRISM elements	Anticipated modification/development focus			
		Intervention modifications	Training	Supervision	Other^a^
Intervention perspectives—organization	Perceived ease of use and usability	x	x	x	
	Perceived adaptability	x		x	
	Ability to observe results	x		x	
	Burden on frontline staff workload and cost	x	x	x	
	Barriers of frontline staff for delivery and access	x	x	x	
	Relevance to frontline staff scope of practice	x	x	x	
	Flexibility with frontline staff tasks	x			
	Perceived familiarity of intervention by organization staff		x	x	
	Awareness of the intervention within the organization		x		x
	Organization staff agreement with intervention focus, processes, and outcomes	x			
	Perception of frontline staff to achieve anticipated outcomes through intervention delivery		x	x	
	Inertia of previous practice		x	x	
	Frontline staff self-efficacy and motivation to deliver intervention		x	x	
	Perceived ability of intervention to engage staff and clients	x	x	x	x
	Organization readiness for the intervention				x
	Alignment with organization mission and change capacity	x			x
	Need for inter-department coordination	x			x
	Strength of evidence base for clinical target area		x		
Intervention perspectives—client	Perception of intervention’s ability to benefit client regardless of clients’ stage of change	x			
	Perception of intervention’s ability to provide client choices	x			
	Addresses barriers to client’s ability to follow advice and set collaborative goals/action plans	x			
	Seamless transition between program elements	x			x
	Intervention addresses barriers to service use and access	x			x
	Minimizes client burden	x			
	Intervention ability to set collaborative goals and action plans with clients	x			
	Ability to provide client feedback on successes and failures	x			
	Concerns about confidentiality	x			x
External environment	Community resources for referrals	x	x	x	x
	Fiscal and regulatory requirements	x			x
	Pay or satisfaction	x			x
	Competition among other organizations	x			x
Recipient characteristics—organization	Use of existing staffing and capacities	x	x	x	x
	Clinical risk management policies	x	x	x	x
	Supervision structure			x	
	Culture of “risk-taking”				x
	Organizational health	x	x	x	x
	Management support and communication				x
	Shared goals and cooperation				x
	Data and decision support tools				x
Recipient characteristics—client	Mental health needs	x	x	x	
	Knowledge and beliefs	x	x	x	
	Demographic characteristics	x	x	x	
	Competing demands	x	x	x	
Implementation infrastructure	Performance data				x
	Dedicated implementation team				x
	Adopter training and support		x	x	x
	Relationship and communication between adopters and bridge researchers				x
	Adaptable protocols and procedures	x			
	Facilitation of sharing of best practices				x
	Plan for sustainability				x

^a^Marketing with organization, workload restructuring, organizational infrastructure

### The 4Rs and 2Ss for strengthening families program

Finally, this study utilizes the 4Rs and 2Ss for strengthening families program (4R2S) to reduce child behavior difficulties as an example EBP (also known as “Multiple Family Groups for Children with Disruptive Behavior Disorders”). This program is currently listed on the Substance Abuse and Mental Health Services Administration’s (SAMHSA) National Registry of Evidence-based Programs and Practices (NREPP; http://nrepp.samhsa.gov/ProgramProfile.aspx?id=41#hide1). 4R2S consists of weekly sessions of multiple family groups (six to eight families) convened over 4 months, where engagement is promoted via extensive phone outreach, childcare, transportation expenses, and a meal provided at each session. Core treatment components integrate two decades of research regarding (1) caregiver engagement in child mental health treatment as well as (2) behavioral parent training and family therapy strategies that address empirically supported family-level influences on disruptive behavior disorders (see Table 2). This intervention is an ideal example of an EBP to use in this study as 4R2S (1) has already been successfully delivered using facilitator teams which include at least one mental health clinician and a non-professional parent advocate (parent consumers of child mental health services) [43–48]; (2) prioritizes engagement and retention of low-income, minority families by targeting logistical (i.e., lack of transportation and childcare, conflicting demands), and perceptual barriers (i.e., stigma) to service use [49–51]; (3) is manualized for straightforward delivery by community-based providers; (4) exemplifies similar EBPs well-known to reduce child behavior difficulties; and (5) does not require specialized placement (i.e., multidimensional treatment foster care [52]) or costly space/equipment requirements (i.e., parent-child interaction therapy [13]).

**Table 2:** Summary of empirically supported family-level influences on ODD and CD

4R2S target	Empirically supported family/parent skill	4R2S goals
Rules [79–87]	Family organization	Clarify rules, consequences, rewards
	Consistent discipline	
Responsibility [81, 87, 88]	Family interconnectedness	Clarify responsibilities, expectations, supports needed, contributions acknowledged
	Positive behavioral expectancies	
Relationships [88–93]	Family warmth	Schedule for positive family interaction
	Within family support, time spent together	
Respectful communication [85, 94, 95]	Family communication, family conflict	Listening and talking skills for parents and children
Stress [50, 51]	Parenting hassles and stress, life events	Identification of stressors undermining family change, promotion of positive exchanges
Social support [49, 50]	Social isolation	Within family and external support plan

Moreover, 4R2S has demonstrated benefits for child behavioral difficulties, functional capacities, and family processes [43, 45–48, 53, 54] in a recently completed effectiveness trial involving a sample of 320 low-income, predominantly minority (Black/African American, Latino) adult caregivers and children (ages 7–11) evidencing oppositional defiant disorder or conduct disorder. Forty percent of participant caregivers indicated having prior or current child welfare involvement at baseline. Compared to participants receiving services as usual (e.g., individual therapy, family therapy), at post-test, participants reported significantly reduced child disruptive behavior difficulties (Cohen’s *d* = 0.35) and increased child social skills (Cohen’s *d* = 0.32). At 6 months’ follow-up, participants who received 4R2S continued to report significantly reduced child behavior difficulties (Cohen’s *d* = 0.34), as well as decreased functional impairment on peer relations (Cohen’s *d* = 0.27), when compared to participants receiving services as usual [43, 53]. Families involved in child welfare services within this sample manifested similar rates of attendance compared to families not involved in child welfare services [55]. Moreover, families involved in child welfare services who received 4R2S reported significantly reduced child behavior difficulties (Cohen’s *d* = 0.61), and functional impairment on peer relations (Cohen’s *d* = 0.44) at 6 months’ follow-up, when compared to child welfare-involved families receiving services as usual [56].

### Primary objectives

The overall aim of the current study is test the feasibility of shifting a mental health intervention from specialized child mental health services to community-based child welfare organizations.

#### Objective 1

The first objective of this study uses a task-shifting framework and PRISM to modify the content, training, and supervision of 4R2S for delivery by child welfare caseworkers in a CBO providing placement prevention services to families and their children with behavior problems. Additionally, task-shifting and PRISM will guide any changes to existing CBO caseload structure and workload in order to promote successful implementation of the modified 4R2S.

#### Objective 2

The second objective of this study is to assess the feasibility and acceptability of the modified 4R2S in a CBO providing placement prevention services.

## Methods/design

### Phase I (objective 1)

#### Participants and settings

To accomplish objective 1, relevant stakeholders invited to consult on the collaborative advisory board (CAB) will include *n* = 2 caseworkers, *n* = 2 supervisors, *n* = 2 agency administrators from CBOs providing placement prevention services, and *n* = 3 parent consumers of placement prevention services. Additional CAB members will include research staff who have had extensive clinical experience conducting the 4R2S intervention. Specifically, the CAB will meet regularly over a period of 4–6 months to modify the 4R2S intervention to create guidelines for ensuring intervention quality and feasibility based on existing task-shifting literature [27, 29–32, 34, 35, 57].

#### Modification of the 4R2S intervention

The CAB will be charged with (1) modifying the existing 4R2S intervention for delivery by CBO caseworkers and identifying any potential changes needed to be made within the CBO to support implementation as well as (2) developing a protocol for training and (3) a mental health supervision framework of CBO caseworkers. This process will be informed by the task-shifting framework (see Fig. 1) and the following PRISM domains: intervention perspectives (e.g., perceived burden, usability/adaptability, barriers), recipient characteristics (e.g., existing staffing capacities, child/family mental health needs, competing demands and barriers), and external environment (e.g., child welfare authority performance indicators, community resources). Research staff will take detailed written field notes of the CAB process (e.g., decision-making, final products) and summarize raw written notes into a structured observation guide [58, 59] with pre-specified headings based on task-shifting strategies as well as PRISM domains and elements (e.g., domain I: considering the intervention from the organization’s perspective) to assist with making subsequent modifications to the intervention, training, and supervision.

CAB sessions are structured to first provide orientation to the overall study. Strategies include team-building exercises and didactic review of study background and procedures as well as didactic and experiential training in the initial 4R2S intervention curriculum sessions. CAB members are subsequently instructed to provide verbal and written feedback that addresses task-shifting strategies and PRISM domains and elements for each session. Secondly, research staff will engage CAB members in a series of discussions focused on addressing the overall 4R2S intervention structure and processes, implementation into the CBO context, as well as caseworker training and supervision. Discussion points are informed by task-shifting strategies and PRISM domains focused on intervention perspectives, recipient characteristics, and external environment. Finally, CAB members will review and provide feedback on all research materials (e.g., recruitment materials, surveys, focus group guides) to ensure readability and acceptability for potential participants.

Subsequent modifications to the 4R2S intervention, training, and supervision will incorporate CAB feedback, methods, and findings from prior studies [43, 44, 53–55, 60] as well as collaboration with 4R2S developers to ensure core concepts and processes are maintained during the modification process. Recommendations from the CAB to alter existing caseload and/or workload structures will be negotiated between research staff and CBO administration in order to maximize implementation success.

#### Training for case workers

Training for caseworkers will integrate recommendations from the extant literature on task-shifting [27, 29–31, 34, 35], implementation science [61], and training for EBPs [62, 63]. As a result, training will include a mixture of (1) didactic instruction on child mental health issues and related family factors, engagement and group facilitation skills, and safety protocols for potential medical and psychiatric emergencies; (2) active and participatory learning strategies (e.g., practice, modeling, role-play, and vicarious learning); (3) provision of an easy-to-follow manual; and (4) ongoing supervision by 4R2S experts. As the original 4R2S intervention were co-led with mental health clinicians, many of whom had substantial expertise in group and family work, supervision with caseworkers will initially focus on adherence to the 4R2S intervention model. However, as caseworkers in the current study will not have had prior mental health training, supervision protocols will further incorporate Stoltenberg’s Integrated Developmental Model [64], which will allow supervisors to monitor and adjust the quality of supervision according to supervisee development (i.e., changes in self and other awareness, motivation, autonomy). For example, supervision in the early part of the task-shifted 4R2S intervention may focus on increasing caseworkers’ confidence and motivation, managing anxieties and frustrations, and providing a high level of structure and direction regarding the clinical skills needed to deliver 4R2S (e.g., conducting group-level interventions, managing child and adult behavior within group sessions) as well as adherence to the intervention model. As caseworkers become more proficient and confident, supervision is likely to shift so that caseworkers develop greater understanding of their clients’ world from cognitive and affective perspectives.

Following the completion of all modifications and development of training/supervision, all revised research materials and procedures will be re-submitted for approval at all participating institutional review boards (IRBs).

### Phase II (objective 2)

In phase II, *n* = 4 caseworkers at a participating CBO will be trained to deliver 4R2S to *n* = 20 caregivers/*n* = 20 youth receiving placement preventive services at CBOs and whose children manifest disruptive behavior difficulties. Quantitative and qualitative approaches will be used to gather information from caseworkers, supervisors, agency administrators, and caregivers to assess feasibility and acceptability.

#### Population

This study will consist of four purposively selected samples: *n* = 4 caseworkers, *n* = 4 supervisors, *n* = 2 administrators, *n* = 20 youth with behavior difficulties, and their caregivers (*n* = 20). Sample sizes for caregivers and youth account for an estimated 20 % attrition rate (overestimating the 10 % attrition from the 4R2S effectiveness study [43, 53]), so that each 4R2S group will contain at least eight families. Although larger sample sizes were considered, smaller samples are sufficient in order to obtain detailed data to establish initial feasibility and acceptability. Such information will be subsequently utilized to revise the modified 4R2S prior to testing in a larger-scale study. A purposive sampling strategy was chosen rather than random selection due to the small sample sizes and desire to enroll engaged participants who can provide important information on feasibility and acceptability.

#### Caseworkers inclusion criteria

Eligible caseworkers (age 21 and older) include *n* = 4 caseworkers who are (1) employed in a CBO contracted to provide placement prevention services, (2) English-speaking, (3) have completed a bachelor’s-level degree, and (4) have had no prior mental health training (e.g., post-graduate training and/or employment as a mental health clinician, clinical licensure).

#### CBO supervisors inclusion criteria

Eligible CBO supervisors (age 21 and older) include *n* = 4 supervisors who (1) supervise the caseworkers enrolled in the current study, (2) are English speaking, and (3) employed at the CBO participating in this study. CBO supervisors are not required to have prior mental health training.

#### CBO administrators inclusion criteria

Eligible CBO administrators (age 21 and older) include *n* = 2 administrators who (1) hold an executive leadership role within the CBO participating in the study, (2) are English-speaking, and (3) employed at the CBO participating in this study.

#### Identified child inclusion criteria

Eligible youth include *n* = 20 children (hereafter referred to as “identified child”) whose permanent caregiver receives placement prevention services at a CBO. Multiple methods will be utilized for screening children who manifest a range of disruptive behavior difficulties who might not be selected if relying on a single screening strategy. This study will enroll English-speaking children ages 7–11 (based on original 4R2S effectiveness study criteria) who meet one or more of the following inclusion criteria: (1) caregiver report of the presence of serious disruptive behavior difficulties using the disruptive behavior disorders (DBD) rating scale [65] (e.g., meeting symptom criteria for oppositional defiant or conduct disorders) and (2) history of out-of-home placement in the past year due to the child’s disruptive behavior difficulties. As a result, these youth may include those temporarily placed in foster care but returned to their permanent caregivers at the time of initial consenting.

#### Caregivers inclusion criteria

Eligible caregivers (hereafter referred to as “legal guardian/primary caregivers”) include up to *n* = 20 adult caregivers (age 21 and older) (1) who are receiving placement prevention services, (2) have a child meeting above criteria, and (3) are English-speaking. If more than one caregiver or child is present and willing to participate in the study, they will both be consented, but only one caregiver will be the data reporter for the family. Research staff will record the number of families screened and subsequently enrolled.

#### Exclusion criteria

Potential family (e.g., caregiver, identified child) participants will be excluded from the study if they present with significant cognitive impairment (as reported by CBO staff) that interferes with understanding the informed consent process. Moreover, for participants with emergency psychiatric needs that require services beyond those within an outpatient setting (e.g., hospitalization, specialized placement outside the home), needed care will be secured rather than study participation. However, if participants are able to remain in the permanent home following an emergency psychiatric evaluation, they may be eligible to participate.

### Recruitment and screening procedures

To recruit caseworkers, supervisors, and agency administrators for phase II, research staff will make multiple presentations at staff and management meetings. Visually appealing fliers will be provided to CBO staff to give to potential participant families (identified child, legal guardian/primary caregiver). Additionally, research staff will have a strong on-site presence at the CBO to provide as-needed informational sessions on the study. All interested individuals may contact research staff directly or allow their contact information to be released to research staff. Finally, research staff will provide in-depth information about the study as well as obtain informed written consent (adult participants) and assent (child participants). Once potential family participants (identified child, legal guardian/primary caregiver) have provided written consent, research staff will administer a screening questionnaire to determine if caregivers and youth are eligible for the study. Of those families deemed eligible to participate in the study, research staff will inform them that additional primary caregivers and siblings may attend 4R2S groups, and will need to be consented/assented as well. Legal guardian/primary caregivers can establish individual meetings with research staff in order to consent/assent additional primary caregivers (aged 21 and older, additional caregiver to identified child) and siblings (6–18 years old, sibling to identified child).

### Setting

The proposed setting is a publicly funded CBO offering placement prevention services to families. This is a different CBO than those employing CAB members from phase I, allowing for consideration of a greater diversity of contextual factors than using the same CBO that employed CAB members. Convenience sampling was utilized, such that research staff approached the state child welfare authority regarding implementing phase II, who subsequently disseminated study information to various jurisdictional authorities in the state. After meeting with several representatives from each interested jurisdiction, the current CBO was chosen as the sole research site due their ability to implement the study within the time constraints of the current grant funding. The placement prevention program, called “in home family preservation,” focuses on stabilizing families and reducing the likelihood of foster care entry. This is accomplished through the direct provision, coordination, or referral to services such as counseling, medical and psychological evaluation, parenting classes, and entitlement assistance to families referred as a result of maltreatment allegations, as well as families voluntarily seeking services.

### Measures

Measures and related study constructs are presented in Table 3. In addition to project-developed measures, the proposed study also uses measures that were previously incorporated in the 4R2S effectiveness study [43, 53]. The DBD rating scale assesses if children meet DSM-5 [66] diagnostic criteria for oppositional defiant disorder, or conduct disorder. Psychometrics of the DBD indicate acceptable test-retest reliability before and after treatment (*r* = 0.49–0.61) and good internal consistency (Cronbach’s alpha = 0.82–0.92). As a measure of concurrent validity, the correlation between the DBD and measures of impairment ranges from 0.79 to 0.93 [65, 67]. The Kazdin barriers to treatment (KBT) scale is a useful measure for understanding the specific barriers to accessing treatment. In addition to looking at the overall average score and comparing to the project-defined cutoff levels, individual items which may be most endorsed by caregiver participants (e.g., “My child refused to come to session”) can be examined in order to inform subsequent intervention modification. Internal consistency for the overall score for experimental participants from the 4R2S effectiveness study was high (Cronbach alpha = 0.94) [55]. The Metropolitan Area Child Study (MACS) Treatment Program Satisfaction Scale further provides a quantitative measure of participant acceptability for the task-shifted 4R2S intervention. Among experimental participants from the 4R2S effectiveness study, good internal consistency was noted at the mid-point and immediately following the 4R2S intervention (Cronbach alpha = 0.88–0.95) [55].

**Table 3:** Measurement and analysis of objective 2

Study construct	PRISM domain	Quantitative measure	Sample qualitative questions
Demographics	Recipient	Project-developed survey^a,c,d,e,f^	Not applicable
Organizational readiness	Recipient	Organizational readiness for change (ORC)^a,d,e,f^	How did the characteristics of your agency affect implementation of the modified intervention? ^a,d,e,g^
Feasibility	External environment	CW performance indicators: % of enrolled families whom CW performance indicators for casework contact goals are met within most recent 6 month period ^b,g^	How were you able to meet requirements by external agencies by using the modified intervention?^a,d,e,g^
Feasibility	Recipient	Client characteristics:	What helped or got in the way of delivering the modified intervention as it was designed? ^a,d,e,g^
		Participant flow: % of children/caregivers meeting inclusion criteria among those screened^b,g^	
		Organizational capacities (ability of caseworkers to deliver 4R2S with fidelity): caseworker fidelity ratings^b,h^	
		HF: % of components scored as “partially met” or “fully met”	
Feasibility	Intervention perspectives	Client perspectives	How feasible was it to implement the modified intervention? What were the challenges? What helped? ^a,d,e,g^
		Attendance logs^b,g^	
		HF: % of children/caregivers who attend ≥80–100 % of sessions	
		Kazdin barriers to treatment (KBT), ^c,g^	Please describe things that influenced your decision to participate or not participate in the modified intervention? ^c,g^
		HF: % of participants with average score ≤2	
		Organization perspective	
		Lyons Acceptability, feasibility, appropriateness scale	
		(LAFAS)—feasibility subscale^a,d,e,g^	
		HF: % of participants with average score ≥4	
Acceptability	Intervention perspectives	Client perspectives	What facilitated/hindered your satisfaction with the modified intervention? ^a,c d,e,g^
		Metropolitan Area Child Study (MACS) Treatment Program Satisfaction Scale^c,g^	
		HA: % of participants with average score ≥3	What are the benefits/challenges of using task-shifting to implement EBPs in CW setting? ^a,d,e,g^
		Organization perspective on intervention	
		LAFAS questionnaire—acceptability and appropriateness subscales ^a,d,e,g^	
		HA: % of participants with average score ≥4	
		Evidence-based practice attitude scale^a,d,e,f,g^ (EBPAS)	
		HA: % of participants with average score ≥3 at post-intervention	

Informant: ^a^caseworkers, ^b^research assistants, ^c^caregivers, ^d^supervisors, ^e^administrators. Timing: ^f^pre intervention, ^g^post-intervention, ^h^ongoing

*HF* high feasibility, *HA* high acceptability

Additional measures are also incorporated in the current study. Subscales of the Organizational Readiness for Change (ORC) [68] will measure training needs, adaptability, orientation to change, stress, and efficacy among CBO staff. The Evidence-Based Practice Attitudes Scale (EBPAS) assesses how CBO staff would respond in the future to implementing 4R2S and other EBPs. As reported in a series of articles [69–71], the EBPAS demonstrates moderate to good internal consistency for the total score (Cronbach alpha 0.74–0.79). Finally, the Lyons Acceptability, Feasibility, and Appropriateness Scale (LAFAS) provides a quantitative measure of feasibility and acceptability. No psychometric information is currently available for the LAFAS.

### Research procedures

#### Baseline surveys

All consented and eligible participants will complete baseline surveys which assess for demographic information, attitudes towards evidence-based practice, and organizational readiness for change and training needs (CBO staff only) as well as child welfare involvement history (legal guardian/primary caregivers only).

#### Training caseworkers and supervisors

Existing training methods from prior effectiveness trial and statewide dissemination of 4R2S [43, 45, 53, 60] will be tailored for use with CBO caseworkers. Such methods involve training service providers and at least one supervisor per site. Training for caseworkers and supervisors will be based on procedures and content developed during phase I of this study. Training will cover the 4R2S model’s core competencies (see Table 2), group facilitation and engagement skills, as well as any additional content identified during phase I CAB recommendations, via a mixture of didactic lecture, group discussion, and role-play. Caseworkers and supervisors will complete a 4R2S knowledge and skills test. Caseworkers who do not pass at the 80 % level will be provided as-needed booster training and be allowed to re-take the test. Only those who pass the test will be allowed to be involved in the study. Caseworker training for the task-shifted 4R2S intervention will be tracked via total number of hours, number of attendees, content covered, and written feedback from caseworkers and supervisors for additional modifications.

#### Conducting 4R2S groups

Following training, CBO caseworkers will deliver the refined and adapted 4R2S intervention to families at the CBOs or possibly in the participants’ residence for missed sessions. 4R2S will be delivered such that two groups will be conducted in the participating CBO. However, each group will be facilitated by two different pairs of caseworkers (two groups total, four caseworkers total). Consequently, two new supervisors and one new administrator will recruited for data collection involving the second group. The length of the resulting task-­shifted 4R2S intervention and mode of delivery (multiple family groups only held at CBOs or groups plus home­based service delivery) will depend on guidance from the CAB. At this time, the original 4R2S intervention is held in weekly group sessions involving approximately six to eight families (at least two generations per family present) over the course of 4 months. At each session, families are provided with childcare for younger children (under 6 years old), refreshments, and transportation expenses. Research staff will ensure that all caseworkers have sufficient materials and resources to conduct 4R2S groups. Meals, childcare, and transportation costs will be covered for all participants at each 4R2S session.

During the course of delivering the task-­shifted 4R2S intervention (up to 4 months), all group sessions will be observed via telephone or web-­based video streaming by research staff in order to complete treatment fidelity rating forms and record session attendance. Additionally, caseworkers will participate in weekly 1-h long conference calls led by research staff (including mental health clinicians with advanced training in child mental health treatment and expertise in the 4R2S intervention) for supervision. Following each 4R2S supervision call, research staff will document the quantity and quality of the supervision call as well as any recommendations for intervention, training, or supervision modification. Research staff will also take written notes during these supervision calls in order to provide real-time insights to research staff on implementation progress. Any challenges noted during each call will allow research staff to provide ongoing problem-solving support in order to facilitate implementation success.

#### Post-test assessment

Upon completion of the task-­shifted 4R2S intervention, CBO caseworkers, supervisors, administrators, and legal guardian/primary caregivers will complete quantitative measures (feasibility, acceptability) as well as participate in separate, homogeneous, audio-recorded focus groups (e.g., caregivers only) or individual interviews (see Table 3 for sample questions).

Following completion of post­test assessments (surveys and focus groups/interviews), research staff will record the information extracted from the CBO’s administrative records for each family regarding referral status, associated maltreatment allegations, casework contacts, service goals and services received, prior out-of-home placement and associated allegations.

### Analytic plan

Quantitative and qualitative results will be integrated during analysis to complement each other in a QUANT→QUAL (given fidelity and attendance will be recorded first) design [72].

#### Quantitative measures and analysis

Table 3 presents information on constructs, corresponding PRISM domains, measures, informants, timing, and methods of analyses. Univariate statistics (means, SD, frequencies) as well as percentages of participants reporting project-defined benchmarks for high feasibility (HF) and acceptability (HA) will be computed. The modified 4R2S will be considered feasible and acceptable if the majority of responses (>50 %) exceed the HF and HA benchmarks.

#### Qualitative methods and analysis

All audio-recorded focus group/interviews will be transcribed verbatim. Written transcripts will be reviewed for accuracy. All written transcripts, as well as all other study documentation (e.g., field interviewer notes, supervisor call notes) will be coded and analyzed using methods by Morgan and Kitzinger [73–76]. Specifically, an initial codebook will be developed by drawing together and comparing a priori (e.g., feasibility, acceptability, external environment, perceptions of the intervention, recipient characteristics) and emergent themes. Rapid qualitative assessment strategies will also be incorporated, which involve documenting initial case summaries categorized by a priori codes [77], as well as remaining open to the emergence of new and unexpected themes. Codes across respondents will be compiled based upon the topics of feasibility and acceptability. For example, a content code “barriers” will be included to identify any content reflecting obstacles to utilizing task-shifting or implementing 4R2S. Codes may be modified as new data are analyzed (i.e., dividing “barriers” into multiple codes such as “system barriers” regarding any organizational obstacles to delivery and “caregiver barriers” for any caregiver-related impediments). A report detailing overall experiences and differences will be compiled by respondent type.

#### Mixed-methods integration

Quantitative and qualitative data will be collected sequentially (quantitative followed by qualitative) and analyzed separately, but focused on answering the overarching and specific research questions being addressed. Research staff will integrate both data types at the interpretation stage of analysis. Quantitative data will be visually juxtaposed (see Table 3) next to relevant qualitative themes in order to aid in interpretation of the combined findings. For example, a research question is whether the modified 4R2S intervention is feasible for CBOs providing child welfare services. Both quantitative and qualitative results will be placed side-by-side in order to compare and contrast, thus determining whether they support convergence (i.e., results confirm each other) or expansion (i.e., results generate additional information about what factors promote or limit feasibility and acceptability).

### Ethical approval

Institutional review board (IRB) protocol approvals for this study have been obtained from the University of Maryland, Baltimore and the Maryland Department of Human Resources. Both protocols will be kept current.

## Discussion

Information gathered from phase I written notes will be compiled into preliminary guidelines documenting the process, successes, and “lessons learned” when utilizing task-shifting strategies. To date, main modifications to the 4R2S intervention for implementation in child welfare include: reducing the length of the model to 9–13 sessions, providing optional instructions if sessions are conducted individually with families during home visiting, addressing literacy concerns by enhancing text size and utilizing more visual information in the intervention manual used by families, adding additional intervention and training content requested by the CAB (e.g., confidentiality concerns, access to local parent advocate information, trauma-informed principles, mindfulness exercises, group and child management). Additionally, organizational-level recommended changes included temporary caseload reduction for group facilitators. Findings from phase I will be disseminated in a future manuscript currently under development.

Phase II processes will provide data on initial feasibility and acceptability, as well as solidify methods (e.g., manual, training, supervision, enrollment) to support a larger-scale study testing the effectiveness and implementation success of task-shifting in CBOs. Overall practical and operational issues involved in performing the study (e.g., obtaining multiple IRB approvals, engaging hard-to-reach participants) will also be discussed as part of barriers and challenges to performing this type of research. In the larger-scale study, implementation and sustainability infrastructure will be addressed, as well as average cost estimates across several different CBOs.

It is acknowledged that a single CBO site is a limitation of the current study, as the contextual factors on implementation and outcomes may vary across different organizations. However, the current study is a first step towards understanding initial feasibility and acceptability of implementing the 4R2S intervention in a child welfare context. The planned larger-scale study will build on these findings by using multiple CBO’s, which will ideally be chosen to reflect a range of organizational contexts (e.g., implementation readiness, culture, climate, size). This study innovatively utilizes task-shifting strategies drawn from international public health efforts to scale-up EBPs in low-resource countries. To the authors’ knowledge, task-shifting has yet to be used to implement child mental health EBPs in child welfare settings. An additional innovation involves the use of PRISM to guide the study’s design and evaluation. Refinement of task-shifting and PRISM strategies can add to the growing arsenal of implementation strategies [78] that could be used as components within comprehensive, multi-level approaches to promote successful EBP implementation. The proposed study will provide generalizable knowledge about using methods to facilitate cross-setting implementation for similar EBPs. Such strategies may also provide an innovative way to increase EBP access and reduce costs within transforming child-serving systems.

### Current status of study

To date, all phase I activities have been completed, all appropriate IRB approvals have been obtained, and phase II procedures are underway.

## Abbreviations

4R2S: 4Rs and 2Ss for strengthening families program
CAB: collaborative advisory board
CBOs: community-based organization
CW: child welfare
DBD: disruptive behavior disorder (rating scale)
EBPs: evidence-based practices
HA: high acceptability
HF: high feasibility
PRISM: Practical, Robust, Implementation, and Sustainability Model
